# Structural and Functional Analysis of the Escherichia coli Acid-Sensing Histidine Kinase EvgS

**DOI:** 10.1128/JB.00310-17

**Published:** 2017-08-22

**Authors:** Hrishiraj Sen, Nikhil Aggarwal, Chibueze Ishionwu, Nosheen Hussain, Chandni Parmar, Mohammed Jamshad, Vassiliy N. Bavro, Peter A. Lund

**Affiliations:** a School of Biosciences, University of Birmingham, Birmingham, United Kingdom; b School of Biological Sciences, University of Essex, Colchester, United Kingdom; Rutgers University-Robert Wood Johnson Medical School

**Keywords:** Escherichia coli, acid resistance, histidine kinase, periplasm, signal transduction

## Abstract

The EvgS/EvgA two-component system of Escherichia coli is activated in response to low pH and alkali metals and regulates many genes, including those for the glutamate-dependent acid resistance system and a number of efflux pumps. EvgS, the sensor kinase, is one of five unconventional histidine kinases (HKs) in E. coli and has a large periplasmic domain and a cytoplasmic PAS domain in addition to phospho-acceptor, HK and dimerization, internal receiver, and phosphotransfer domains. Mutations that constitutively activate the protein at pH 7 map to the PAS domain. Here, we built a homology model of the periplasmic region of EvgS, based on the structure of the equivalent region of the BvgS homologue, to guide mutagenesis of potential key residues in this region. We show that histidine 226 is required for induction and that it is structurally colocated with a proline residue (P522) at the top of the predicted transmembrane helix that is expected to play a key role in passing information to the cytoplasmic domains. We also show that the constitutive mutations in the PAS domain can be further activated by low external pH. Expression of the cytoplasmic part of the protein alone also gives constitutive activation, which is lost if the constitutive PAS mutations are present. These findings are consistent with a model in which EvgS senses both external and internal pH and is activated by a shift from a tight inactive to a weak active dimer, and we present an analysis of the purified cytoplasmic portion of EvgS that supports this.

**IMPORTANCE** One of the ways bacteria sense their environment is through two-component systems, which have one membrane-bound protein to do the sensing and another inside the cell to turn genes on or off in response to what the membrane-bound protein has detected. The membrane-bound protein must thus be able to detect the stress and signal this detection event to the protein inside the cell. To understand this process, we studied a protein that helps E. coli to survive exposure to low pH, which it must do before taking up residence in the gastrointestinal tract. We describe a predicted structure for the main sensing part of the protein and identify some key residues within it that are involved in the sensing and signaling processes. We propose a mechanism for how the protein may become activated and present some evidence to support our proposal.

## INTRODUCTION

Among the many different two-component systems (TCS) that detect signals and regulate gene expression in bacteria, one intriguing group is typified by histidine kinases (HKs) that possess one or more periplasmic Venus flytrap (VFT) domains (1–3). VFT domains are found in proteins across all kingdoms. In bacteria, they are largely a feature of amino acid binding proteins, particularly periplasmic binding proteins (PBPs) that bind solutes for subsequent import into the cell via transporters. Such proteins have been classified in various ways based on their structure, and such classifications correlate well with the type of solute that is bound (4–6). VFT domains contain two globular subdomains or lobes containing mostly alternating alpha-helices and beta-sheets that flank the binding site of the ligand, and they can exist in open or closed states. Transition in the equilibrium between the two states is caused by the binding of the ligand, which causes “closure” of the trap (7). Although mostly associated with PBPs, VFT domains are also found on many membrane-bound HKs, with the number ranging from one to five. Many of these HKs are members of TCS in pathogens that are associated with virulence, and their ligands are unknown (1, 3).

The best characterized of this large group of HKs is the BvgS protein found in the bordetellae. This protein regulates the expression of virulence factors, including toxins and adhesins, via the response regulator BvgA, and it has a large number of genes in its regulon (8). The natural ligand for BvgS is unknown, and while it is constitutively active in cells grown at 37°C, it is switched off by low temperature or exposure to high concentrations of sulfate ions or nicotinic acid (reviewed in reference 1). The protein has a molecular mass of approximately 135 kDa, of which nearly 60 kDa is in the periplasm, linked to the cytoplasmic part by a single transmembrane helix. It is dimeric, and the structure of the periplasmic domain has been solved (2, 3), revealing two VFT domains in each protomer, wrapped around each other with one in the open and one in the closed conformation. Downstream of the transmembrane helix is a PAS domain, a common feature of many regulatory proteins, including HKs, which in turn is followed by phospho-acceptor (carrying the target histidine residue, H729) and kinase domains. Unusually compared to most TCS kinases, the phosphate is transferred to an aspartate, D1023, in an internal receiver domain before being transferred to a final histidine, H1172, in a histidine phosphotransfer domain (9–11). From here, it is ultimately transferred to the receiver aspartate in BvgA. This pattern of internal phosphotransfer in so-called unorthodox HKs has been proposed to make them more sensitive to the signal within a critical range and more robust against noise (12, 13). A detailed mechanism relating movement of domains in the periplasm to a subsequent alteration in the dynamics of a coiled-coil region immediately after the PAS domain has recently been proposed, which may be generic to all proteins with this domain structure (14).

In Escherichia coli, the closest homologue to BvgS is the EvgS HK, which shares the same domain organization. The *evgS* gene and the gene for its cognate response regulator, *evgA*, were first cloned as multicopy suppressors of a Δ*envZ* mutation (15). Early studies showed that EvgA binds to the region upstream from the *evgAS* operon and in doing so regulates both the operon and the divergently transcribed *emrKY* genes, which encode a multidrug efflux pump (16, 17). Overproduction of EvgA leads to elevated resistance to multiple drugs and toxins, which was attributed to upregulation of the EmrKY and the YhiUV (subsequently renamed MdtEF) transporters (18–20). EvgA overproduction also leads to the expression of an acid resistance phenotype in exponential phase, a finding that enabled the identification of several new genes implicated in acid resistance in E. coli, including the one encoding the central regulator GadE, as well as the elucidation of part of the complex network of interactions governing expression of the glutamate-dependent acid resistance mechanism of E. coli, AR2 (also called the GAD system) (21–24). Deletion of either *evgA* or *evgS* leads to the complete loss of acid induction of all the key genes in the AR2 network (25, 26). Evolution experiments with selection for long-term survival at pH 2.5 in exponential phase led repeatedly to the isolation of mutations that rendered EvgS constitutively active at pH 7, and mutants of EvgS selected for their constitutive activation of an *emrK-lacZ* fusion also showed an acid resistance phenotype (17, 25, 27). Thus, the *evgAS* regulon is important both in acid resistance and in drug efflux in E. coli.

Comparative analysis of the transcriptomes of strains expressing different constitutively active EvgS proteins shows a considerable overlap of upregulated genes, including the genes in the acid fitness island, genes adjacent to the *evgAS* operon (involved in drug efflux and in oxalate decarboxylation), and components of cytochrome *bd-II* oxidase and periplasmic hydrogenase. Activation of the regulators GadE, GadX, GadW, and YdeO by phosphorylated EvgA has a complex and central role in activation of the main structural genes of the glutamate-dependent acid resistance system (*gadA*, *gadB*, and *gadC*) plus other genes in the acid fitness island that encode periplasmic chaperones and the outer membrane lipoprotein Slp, as well as a range of unlinked genes, including those for the flagellar protein FliC and the large and small subunits of glutamine synthetase (25, 26, 28, 29). In pathogenic E. coli O157:H7, the GadE regulon has also been shown to include the Ler protein, which itself represses many of the genes in the locus of enterocyte effacement (LEE) pathogenicity island, and mild acidification leads to downregulation of LEE gene expression (30). Thus, the EvgAS TCS has the potential, through its function in regulation of GadE and other central acid response regulators, to have important roles in E. coli pathogenicity. Indeed, overproduction of EvgA has been found to reduce expression of the components of the type three secretion system encoded by the LEE region (31), while *evgS* mutants are attenuated in an avian lung infection model with an avian-pathogenic strain of E. coli (32).

Our previous study on EvgS showed that many different mutations in the PAS domain led to constitutive activation of the protein and that deletion of part of the periplasmic domain led to loss of inducibility by low pH but no loss of activity of these constitutive mutants (27). We proposed a model based on these data whereby detection of the inducing signal takes place in the periplasmic domain. This results in transmission of an activating signal across the inner membrane, which in turn leads to the weakening of subunit interactions in a tight and inactive dimer of EvgS. By this model, the active form is proposed to be a loose dimer, and the activating mutations in the PAS domain are proposed to also weaken interactions in the dimer, thus mimicking the activation of EvgS that normally occurs only on external mild acidification. Here, we have constructed a model structure of the EvgS periplasmic domain, based on the published BvgS periplasmic domain structure, to guide site-directed mutagenesis of residues in the periplasmic domain. We report the identification of key periplasmic residues needed for low-pH induction of EvgS. We also show that the cytoplasmic domain alone can still mediate some pH-dependent gene expression, and we propose from this finding a more complete model for how EvgS may function, with some supporting biochemical evidence.

## RESULTS

### Prediction of the structure of the EvgS periplasmic domain: do amino acids influence induction of EvgS activity?

The BvgS and EvgS proteins share a common domain organization and exhibit a high degree of sequence similarity (28.8% identity). Both are examples of unconventional HKs which have internal receiver domains to which the phosphate is initially transferred after autokinase activity has phosphorylated a histidine residue in the HK domain, rather than the more common direct transfer to a response regulator. The phosphate is subsequently passed to a second histidine in the histidine phosphotransfer domain for eventual transfer to an aspartate in the cognate response regulator protein (33, 34). Both proteins have unusually large periplasmic domains, each containing two VFT domains. The recent availability of high-resolution structures of the BvgS periplasmic domains (the isolated VFT2 domain [PDB 3MPK] [2] and the dimeric complete periplasmic portion of the protein [PDB 4Q0C] [3]) allowed us to construct high-fidelity homology models of the EvgS periplasmic domain. The available structure of BvgS covers residues 33 to 544 of the mature protein, corresponding to residues 25 to 538 of E. coli EvgS (NCBI reference sequence WP_072282315), after which the protein is predicted to cross the inner membrane via a single transmembrane helix. These periplasmic regions have 25% identity (122/497 residues) and 46% similarity, with only three single-residue gaps and one two-residue gap in the alignment produced using MAFFT (see Fig. S1 at http://epapers.bham.ac.uk/3021/), allowing unequivocal assignment of all the secondary structure elements and the building of reliable homology models. A range of models was created using the I-TASSER modeling suite, yielding models with a C-score value between +0.75 and +0.95, indicative of high confidence and strong predictive power. The highest overall scoring model was used for analyzing the structural properties of EvgS and making selections for the residues to be targeted by point mutagenesis as described below.

**FIG 1:**
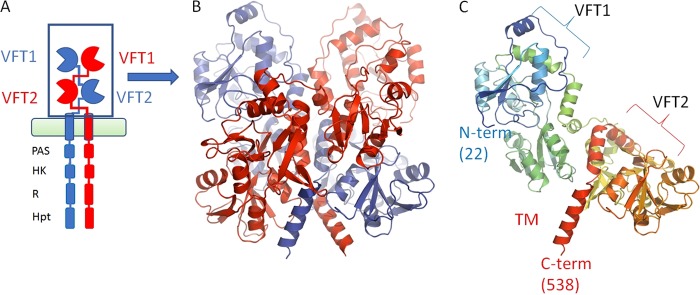
Schematic of EvgS showing the domain organization and the modeled section discussed in the text. (A) Domain organization of EvgS, showing the membrane and intracellular kinase domains. (B) The periplasmic domain is composed of two Venus flytrap domains, VFT1 and VFT2, which dimerize. The different chains are colored red and blue, respectively. (C) A single protomer showing the domain organization and colored in rainbow from N terminus (blue) to C terminus (red). The homology model presented covers residues 22 to 538 of the full-length protein.

As we used the BvgS structure as the primary model template, the predicted EvgS periplasmic domain is compatible with the dimeric organization of BvgS, having two VFT domains in each protomer, with a 2-fold symmetry axis parallel to the long axis of the dimer. The peptide chain shows broadly the same path in each protomer and crosses twice between the sides of the dimer before passing into the alpha-helix, which is predicted to cross the membrane (Fig. 1A and B). The conformation of the EvgS model is also consistent with the state of the BvgS in the crystal structure, and correspondingly each monomer in the model structure contains the two distinct VFT domains, with VFT1 in an open conformation and VFT2 in a closed conformation. While the conformation presented is highly probable and is compatible with the EvgS sequence based on its close homology to BvgS, one needs to keep in mind that this conformation is chosen due to model bias and is impossible to validate directly at this point. Figure 1C shows a single protomer to make the arrangement of the two domains in the complex clearer. A detailed description of the characteristics of the full structure can be found in reference 3.

To see what biological insights we could get from homologous structures, we searched the Protein Data Bank (PDB) database for the closest structural matches to the EvgS structure other than the template BvgS structure itself. From this search we identified PDB 4PRS and 4PSH, which are structures of an arginine binding protein that works with an ABC transporter from the extreme thermophile Gram-negative bacterium Thermotoga maritima (35) without and with a bound arginine, respectively. The holo-structure reveals a well-defined arginine moiety coordinated by a number of residues, including R82 (as numbered in the PDB file), and closely resembles the VFT2 domains of both BvgS and EvgS (see Fig. S2 at http://epapers.bham.ac.uk/3021/). The same type of coordination to bound amino acids is seen in other examples, which all show significant structural homology with the VFT2 domain, including 3VVE (a protein involved in lysine transport in Thermus thermophilus) (36), 4H5F (an arginine binding ABC transporter from Streptococcus pneumoniae) (P. J. Stogios et al., unpublished reference from the PDB entry), and 3LSL (glutamate binding domain of GluA2, human AMPA receptor) (37). The coordinating arginine residue common to all these structures is conserved in both BvgS (R380) and EvgS (R375), although its removal in BvgS has been shown to have no effect on BvgS activity (2).

**FIG 2:**
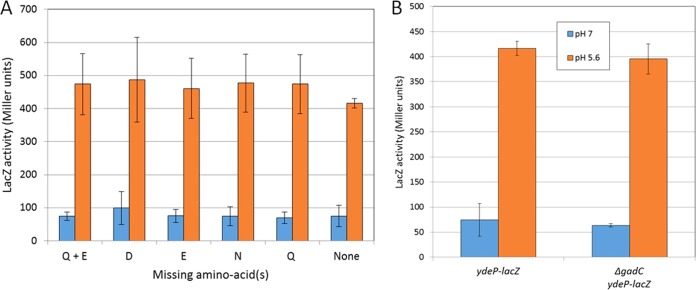
EvgS induction does not require amino acid binding or activity of the GadC glutamate transporter. (A) Activity of the *ydeP-lacZ* reporter at pH 7 and pH 5.6 in the absence of selected amino acids (the induction ratios for all amino acids are shown in Fig. S4 at http://epapers.bham.ac.uk/3021/). (B) Activity of the *ydeP-lacZ* reporter at pH 7 and pH 5.6 in the presence and absence of the GadC antiporter.

The binding of glutamate (shown in Fig. S3 at http://epapers.bham.ac.uk/3021/) was of interest, as EvgS activates the AR2 system in E. coli, which requires the import of external glutamate as a substrate via the GadC antiporter (28, 29), and this led us to speculate that binding of glutamate might be required for EvgS activation. We tested this possibility by assaying EvgS-mediated induction of a *ydeP-lacZ* promoter fusion as described in Materials and Methods but with different amino acids or groups of amino acids omitted from the growth medium. No effects on induction were seen when different combinations of glutamate, glutamine, aspartate, or asparagine were omitted from the growth medium (Fig. 2A). Data for other groups of amino acids are summarized in Fig. S4 at http://epapers.bham.ac.uk/3021/ and also show no effect. We also checked to see whether glutamate transport might be necessary for EvgS activation, as there are precedents for TCS being activated by the transport of their ligand (for a recent review, see reference 38). To do this, we deleted the gene for the GadC antiporter from the reporter strain by P1 transduction. As shown in Fig. 2B, this deletion also had no effect on EvgS-mediated induction of *ydeP-lacZ*. Thus, despite the close structural link of EvgS to the amino acid binding proteins, we have detected no amino acid influence by either binding or transport over the activation of EvgS, although we cannot rule out the possibility that one or more amino acids bind to VFT2.

### Potential roles of periplasmic histidine residues in EvgS activity.

The VFT domain closer to the N terminus of the protein, VFT1, was also investigated by initially looking for structural clues. We superimposed the arginine binding region of ArgBP as defined for 4PSH over this domain. Structural similarity could be seen, but the root mean square deviation (RMSD) in this case was over 4 Å, as opposed to 2.3 Å for the overlay with VFT2, showing that VFT1 is more divergent structurally from ArgBP. When we analyzed VFT1 from the model and ArgBP more closely, two points were noted. The first was that there is no conserved arginine corresponding to R82 (the centrally important residue which coordinates the carboxyl ring of the ligand in all the amino acid binding proteins) in this domain. Instead, EvgS has T127 and S128 in the corresponding positions, which would make it impossible to coordinate a canonical amino acid in VFT1. The second is a striking cluster of three histidine residues which protrude into the binding cleft of VFT1 (H63, H106, and H124). Both of these features are shown in Fig. S5 at http://epapers.bham.ac.uk/3021/. Although the histidines do not sterically clash with the position that a bound amino acid would take, the VFT1 domain is in its open conformation in this structure, whereas if a ligand were to bind, it would change to the closed conformation, potentially increasing the impact of this histidine triad on the VFT1 binding domain. Taken together, and allowing for the uncertainty associated with modeling bias, these two observations make it less likely that an amino acid-like ligand might stably bind to the VTF1 domain.

**FIG 3:**
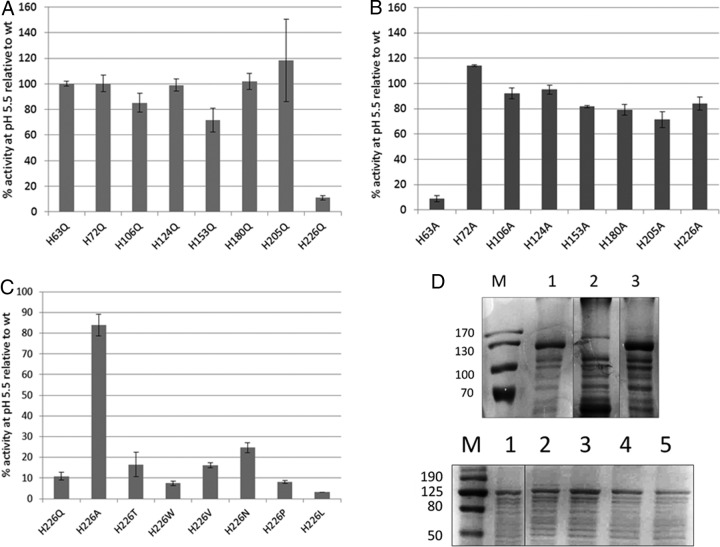
(A to C) Induction at pH 5.6 of EvgS proteins mutated at different histidine residues in the periplasmic domain, expressed as a percentage of the induction seen with wild-type EvgS. (A) Histidine to glutamine. (B) Histidine to alanine. (C) H226 to multiple different amino acids. (D) Top gel, membrane fractions from cells expressing wild-type EvgS (lane 1), EvgS H63A (lane 2), or EvgS H226Q (lane 3). Bottom gel, membrane fractions from cells expressing wild-type EvgS (lane 1), EvgS H226A (lane 2), EvgS H226V (lane 3), EvgS H226W (lane 4), or EvgS H226P (lane 5). Markers (lane M) are labeled in kilodaltons. Vertical lines on the gel images show where additional lanes run on the original gels have been spliced out for clarity.

The histidine triad presents an interesting possibility as a potential pH sensor. Histidine residues are good candidates for detecting changes of pH due to their protonation under mild acid conditions. The optimum pH for induction of EvgS activity has been shown to be around 5.5 to 5.7 (26, 39), and the pK_a_ of histidine residues can often fall in this range (40). To test this possibility, particularly for the histidine triad but also for all the other histidine residues in VFT1, we constructed mutations where we individually replaced all the histidines in the VFT1 domain with alanine or glutamine and measured their impact on EvgS-induced *ydeP-lacZ* activity. The data (Fig. 3A and B) show that all histidines except H63 could be changed to alanines without loss of induction and that all except H226 could be changed to glutamines without loss of induction.

One plausible reason for loss of induction is that the mutation leads to a failure to correctly fold the protein or to loss of targeting of the protein to the membrane. To check whether this possibility was an explanation for our data, we prepared crude membrane fractions of strains expressing wild-type EvgS and the two noninducible mutants EvgS H63A and EvgS H226Q and compared them on SDS-PAGE. As can be seen in Fig. 3D (top gel), no band corresponding to EvgS H63A could be detected, whereas EvgS H226Q was clearly visible in membrane fractions, as was the wild-type protein. This result shows that loss of induction of H63A is almost certainly a trivial consequence of the absence of the protein, but loss of induction for H226Q must have a different explanation.

The fact that EvgS H226Q cannot be induced at low pH but EvgS H226A can suggests that while this residue is likely to play a critical role in some aspect of signal detection or transduction, protonation is not involved in this process. To investigate this further, we made another series of mutations at this position (to T, L, V, W, N, and P) and confirmed that all of the mutants except H226A showed a significant decrease in inducibility at pH 5.6 relative to that of the wild-type protein (Fig. 3C). Several of these mutants were tested for EvgS protein levels in membrane fractions, and all still produced a visible protein band (Fig. 3D, bottom gel), confirming that this residue is crucial for EvgS activity.

None of the mutations in any of the histidine residues in the potential triad led to loss of inducibility of EvgS, with the trivial exception of H63A, which led to loss of the protein. To test whether functional redundancy might be built into this triad, we constructed all possible pairwise combinations of histidine to glutamine and the triple H63Q H106Q H124Q mutant. As shown in Fig. S6 at http://epapers.bham.ac.uk/3021/, all of these mutants still showed the same level of induction as the wild-type EvgS, ruling out any role of this triad in detection of the inducing signal.

### Identification of a potential interaction between H226 and P522 at the top of the predicted transmembrane helices.

**FIG 4:**
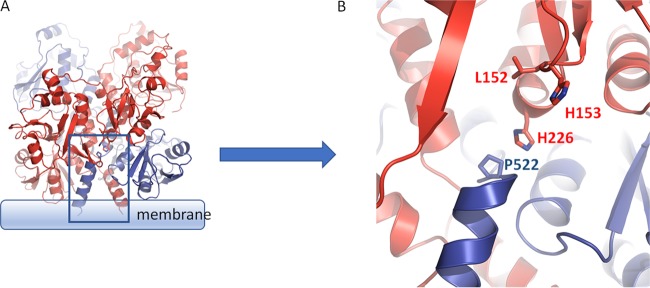
Residues in the interdomain of the VFT1 and VFT2 interface are crucial for EvgS activation and may be involved in signal transduction involving Pro522. (A) A general view of the region discussed. (B) Image rotated and zoomed in to show individual amino acid side chains.

**FIG 5:**
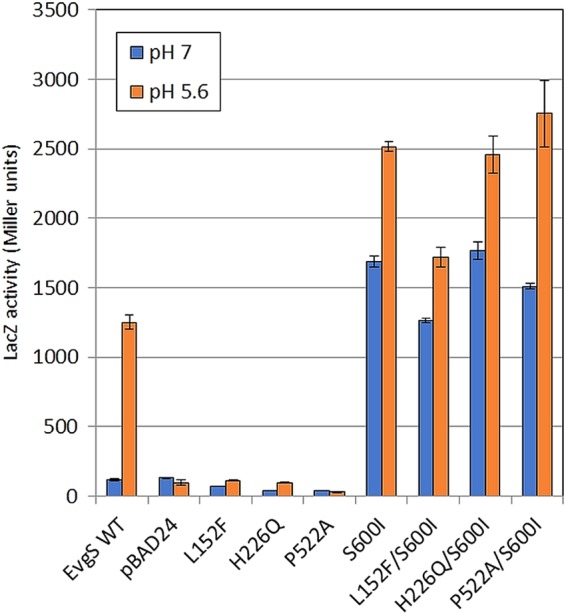
Impact of the periplasmic L152F, H226Q, and P522A mutations on EvgS-mediated induction of the *ydeP-lacZ* reporter at pH 5.6, alone and when combined with the constitutively activating S600I mutation.

We examined the position of the critical H226 residue in the modeled structure of EvgS. H226 is one of two histidines (the other being H153) that protrude in an antenna-like fashion from each monomer when those are considered in isolation. In the context of the dimer, however, H226 is deeply embedded in the structure and makes several possible intraprotomer and interprotomer contacts which may relate to its function. In particular, we have noted that H226 is located in close proximity to a proline residue (P522) which we predict is likely to cap the predicted transmembrane helix of the opposite protomer (Fig. 4), though it should be noted that the confidence of the model in this region (positions 521 to 525) is somewhat lower, with a gap in the template (BvgS) alignment due to an extra residue in the EvgS sequence after position 523. P532 in BvgS clearly caps alpha-helix 17, which leads to the transmembrane domain. If we extend the alignment from the transmembrane domain backwards, the residue P522 in EvgS is found before an extra predicted helical turn, to cap the corresponding helix 17. The proximity of P522 to a number of other residues suggests that it could play a role as the transducer of conformational switches both from intraprotomer transitions (e.g., opening and closing of VFT domains) and interprotomer transitions. Based on these predictions and to test whether P522 is indeed also needed for induction of activity of EvgS, we constructed a P522A mutant and analyzed its inducibility. We also noted that another residue that maps to the same locality and that protrudes from each monomer toward the neighboring protomer is L152, adjacent to H153, which has previously been mutated to F and identified as being required for activation of EvgS (39). We reconstructed this mutant and tested it in the same analysis as a positive control. The results (Fig. 5) confirmed that none of these mutants showed any detectable activity above background at pH 7 or pH 5.6. Due to difficulties in producing reproducible results when trying to detect these mutated proteins in membrane fractions, we added the activating mutation S600I to the cytoplasmic PAS domain of each mutant to see if they regained activity. S600I is one of the mutations whose presence makes EvgS constitutively active at pH 7, and we have previously shown that if S600I is introduced into an EvgS mutant that has been rendered noninducible at pH 5.6 by deletion of part of the periplasmic domain, the protein shows constitutive activity (27). Thus, by introducing the S600I mutation into EvgS proteins that carry single-amino-acid mutations in the periplasmic domain, we can distinguish mutated proteins that may be misdirected, misfolded, or degraded (and which hence will have no activity) from those that do reach the membrane but are not activated as normal by low external pH. In each of the three cases tested (L152F, H226Q, and P522A), the addition of the S600I mutation to EvgS that contained these mutations in the periplasmic domain led to levels of activity at pH 7 which were very similar to that of EvgS S600I. We also noted that in EvgS S600I and in each of the three double mutants, further induction (an average of 1.51-fold [standard deviation, 0.21]) was seen when cells were incubated at pH 5.6 (Fig. 5). The periplasmic mutations thus do not compromise the potential for EvgS to be active, but they do render it noninducible by low pH. However, they do not alter the constitutive activity at pH 7 or the inducibility at pH 5.6 of EvgS S600I when they are introduced into it, a point that is considered in the next section. Possible roles of the H226 and P522 residues are considered further in Discussion.

### Cytoplasmic EvgS shows pH-dependent dimer formation *in vitro*.

The inducibility at pH 5.6 of EvgS S600I, whether the mutation is present on its own or combined with a mutation in the periplasmic domain that blocks induction, could result from the detection of a signal associated with low pH taking place at the membrane or in the cytoplasm. The first explanation is rendered less likely by the fact that a membrane-anchored form of EvgS lacking the whole periplasmic domain has already been studied in the same assay system as used here and shows no activity at either pH 7 or pH 5.6 (39). We therefore decided to express the entire cytoplasmic region of EvgS with no membrane attachment to see whether it still showed any pH-dependent increase in activity. The construction and expression of this protein, which we call EvgS-Cyt, is described in Materials and Methods.

**FIG 6:**
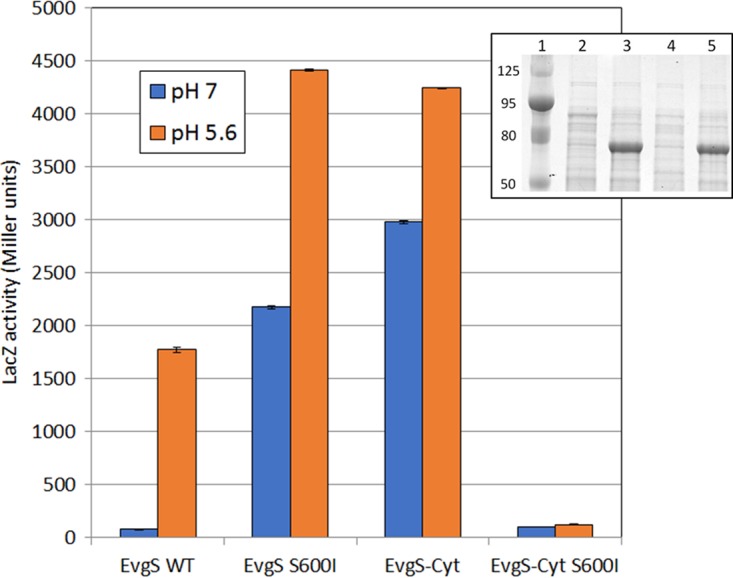
Comparison of activities of wild-type EvgS and EvgS-Cyt without and with the activating S600I mutation in the cytoplasmic PAS domain. The inset shows a 12.5% SDS-polyacrylamide gel of crude extracts from the reporter strain expressing EvgS-Cyt (lanes 2 and 3) or EvgS S600I-Cyt (lanes 4 and 5), without and with induction, respectively; lane 1 shows molecular mass markers in kilodaltons.

It is quite common for the cytoplasmic portions of TCS HKs to show constitutive activity when expressed without the transmembrane and periplasmic regions (see, e.g., references 41 and 42), and this proved to be the case with EvgS-Cyt, in that the *ydeP-lacZ* reporter showed high activity at pH 7 in the presence of EvgS-Cyt and the absence of any full-length EvgS. Moreover, as observed with the constitutive mutant EvgS S600I, we saw a further induction of activity when cells expressing EvgS-Cyt were incubated at pH 5.6, supporting the hypothesis that EvgS can sense a cytoplasmic signal that changes when the external pH drops (Fig. 6). Whether this signal is pH itself or something else remains to be determined.

We previously proposed a model to explain the high frequency with which mutations that activate EvgS and map in the PAS domain are found. In this model, we suggested that the nonactive state of EvgS could be a tight inactive dimer. Activation is caused when a detection signal is received which moves the equilibrium of EvgS from a tight, inactive dimer to a weak, active dimer. According to this model, the PAS domain mutants cause constitutive activation at pH 7 by weakening the dimer interactions in the absence of an inducing signal, and consistent with this, many of these mutations map at or close to the predicted dimer interface (27). The model can explain the activation of EvgS signaling when the cytoplasmic domain alone is expressed, as the domain will now have lost those interactions in the periplasmic and transmembrane domains that normally contribute to the tight, inactive state.

In its simplest form, our hypothesis proposes that EvgS can exist in three states: a tight, inactive dimer; a weak, active dimer; and a weaker inactive dimer or monomer, with this third state being seen only when the periplasmic and transmembrane domains are missing and an activating mutation is present in the PAS domain. If this hypothesis is correct, we can predict that introducing a PAS domain mutation (which weakens subunit interactions) into the non-membrane-anchored cytoplasmic domain (in which interactions have already been weakened) would further reduce the degree of association of the subunits of the dimer, potentially to the point that it no longer shows any activity at all. We tested this prediction and confirmed that if the activating S600I mutation is introduced into EvgS-Cyt, all activity and inducibility is lost (Fig. 6) even though the protein is still made and can be clearly seen on an SDS-polyacrylamide gel at the same level as EvgS-Cyt (Fig. 6, inset).

**FIG 7:**
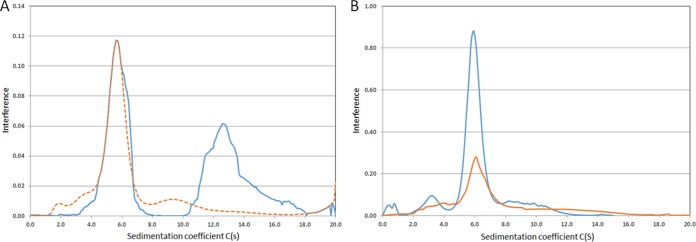
Analytical ultracentrifugation analysis of purified EvgS-Cyt (A) and EvgS-Cyt S600I (B) at pH 7 (blue lines) and at pH 5.6 (orange lines). The pH 5.6 data are plotted as a dashed line in panel A to make the coincidence of the lower-molecular-weight peaks clear. Different peak heights are due to different amounts of protein loaded in AUC cells.

To test our model biochemically, we purified the EvgS-Cyt protein and compared its behavior in an analytical ultracentrifuge when run at either pH 7 or pH 5.6 (Fig. 7A). Strikingly, the protein sediments predominantly as two peaks at pH 7, consistent with a monomer-dimer equilibrium as required by our model. The breadth of the dimer peak and the presence of shoulders suggests that even higher-order structures may form. The mass distribution shows that the molecular mass of the first peak is 81.7 kDa and that of second peak is 163 kDa, quite close to the expected values for monomer and dimer (72.3 kDa and 144.6 kDa, respectively). At pH 5.6, a small amount of the protein aggregates, and the lower-molecular-mass peak becomes somewhat broader, with a clear shoulder at a slightly high molecular mass, although the peak exactly coincides with the peak at pH 7. The higher-molecular-mass peak seen at pH 7 was not visible at pH 5.6. This result is consistent with a weakening of the dimer at the inducing pH, as predicted by our model. When the same analysis was done with purified cytoplasmic domain containing the activating S600I mutation, the analytical ultracentrifugation (AUC) results were practically identical at pH 7 and pH 5.6 (Fig. 7B), with a mass distribution of 76.22 kDa for both peaks, which is very close to the expected value. This result is consistent with our model, where the activating mutations in the PAS domain mimic the effect of low pH by producing a shift in the equilibrium away from the tight, inactive dimer state even at a normally noninducing pH of 7, such that the EvgS protein is now active at neutral pH.

## DISCUSSION

Several obvious questions can be asked for any HK which functions as part of a TCS, and answering these questions for any one kinase will contribute to our understanding of TCS in general, as well as improving our understanding of the particular system that it regulates. The first question usually concerns the nature of the signal that is recognized. In the case of EvgS, the actual signal is not known, and although a reasonable primary assumption is that the protein responds directly to changes in pH, there is no experimental proof for this assumption. Indeed, although several other HKs which are activated by low pH have been successfully studied to identify key residues for signal detection, direct demonstration that pH is the actual signal detected would require approaches such as reconstitution in a proteoliposome system where the pH can be manipulated or direct identification of pH-affected residues by deuterium exchange, and such experiments have not been done to date. In living cells, the consequences of changing external pH will be both significant and multifaceted, particularly given the importance of transmembrane proton gradients in energy metabolism, so proof that pH is the direct signal is hard to obtain. Nonetheless, systematic studies of several other HKs have revealed a range of amino acids that may directly or indirectly detect pH change. In the ArsS kinase of Helicobacter pylori, a specific histidine residue (H94) was shown to be required for pH detection, although other residues were also shown to make a contribution (43). The SsrA HK in Salmonella enterica was shown to require two clusters of histidine residues in the periplasmic domain to detect low pH, with mutations in individual residues having relatively small effects (44). The E. coli CadC protein, which regulates the lysine-dependent AR4 system, is a ToxR-like protein with its C terminus in the periplasm, which has to interact with the lysine transporter LysP at low pH in order to activate gene expression (which as a one-component system it does directly, rather than by interacting with a response regulator protein [45]). Histidines play no role in the pH detection mechanism, which has been shown to rely on a cluster of acidic residues that form a negatively charged patch in the periplasmic region of the protein (46). pH detection is also required by the transporters GadC and AdiC of the E. coli acid resistance systems AR2 and AR3 (both of which function only below pH 6), and it has been recently shown that a tyrosine residue (Y74) is critical for pH sensing in AdiC, a finding that has been confirmed in an *in vitro* proteoliposome transport system (47). Thus, a range of different protonatable residues are used by different proteins as pH detectors.

None of the above examples are of proteins with large periplasmic VFT domains, so they cannot be directly used to identify candidate residues in EvgS. We have not yet succeeded in identifying residues in EvgS which detect low pH in EvgS, and given the large size of the periplasmic part of the protein, systematic mutagenesis of all candidate residues will be a considerable undertaking. It is also the case that EvgS may not directly detect pH at all, but detects some other signal. Indeed, earlier studies have shown that soluble fragments of both EvgS and BvgS were strongly inhibited in their *in vitro* phosphorylation by oxidized ubiquinone, suggesting a possible direct link to the electron transport chain and energy metabolism of the cell (48). However, the work reported here does rule out several candidates for signal detection. Although the triad of histidines in VFT1 was an attractive candidate, mutagenesis rules out a role in signal detection for this cluster, as it does for most of the other histidine residues in VFT1. It remains possible that key histidine residues in VFT2 will yet be found, although nothing in the structure points to any strong candidates. We also ruled out the possibility that amino acid binding in VFT2 modulates EvgS activity, and we showed on a structural basis that amino acid binding to VFT1 is very unlikely. We examined the potential role of GadC activity in the light of systems such as the AR4 system, which requires lysine transport by the LysP protein for activation, but we showed that GadC was not needed for EvgS activation. However, we did identify one histidine (H226) as being important for activation of the kinase. The mechanism for its role is unknown, but the structural model showed it to be close to L152, which already has been identified as being required for activation (39), and to P522, which caps the predicted transmembrane helix. The location of the H226 and L152 residues close to the dimer interface in the modeled structure provides a plausible mechanism whereby a structural change in the periplasmic domain could lead to a movement being transmitted via the transmembrane helix to the cytoplasm, with such movements being intraprotomer or interprotomer. Communication of structural changes from extracellular and periplasmic detector domains to transmembrane helices that rely on the presence of a conserved proline residue at the top of the helix or in the loop joining two consecutive transmembrane helices is seen in numerous receptor structures, such as in the family of pentameric ligand-gated ion channels (e.g., the acetylcholine and gamma-aminobutyric acid [GABA] receptors in humans), where such changes are key parts of the gating mechanism (reviewed in reference 49). The resulting small displacements in the transmembrane helices of the EvgS dimer could activate the cytoplasmic HK activity of EvgS in a way analogous to those reported in a related family of sensory kinases that includes EnvZ (50, 51).

Once the signal has been transmitted across the membrane, it has to produce a structural change somewhere in the cytoplasmic domains of the kinase in order for the kinase activity to be activated (or in the case of inhibitors, for it to be repressed, or for an auto-phosphatase activity, for it to be activated). Significant evidence reported by us and others previously had already pointed to the PAS domain just after the membrane as having a key role in the activation of both EvgS and BvgS. Mutations in this domain that activate EvgS at pH 7 or block the negative effects of the modulators of BvgS are easy to find, which implies that they represent a loss of some aspect of the protein's normal structure rather than a novel functional structure (27). The simplest explanation for this is that they cause weakening of an inactive tight dimer. Histidine kinases are generally found to be active as dimers, and there are precedents for a weakening of the dimer being the mechanism of activation in some cases. For example, the DctB HK found in Sinorhizobium meliloti, which detects and responds to the presence of C_4_ dicarboxylic acids, is believed to proceed to an activated state by weakening of interactions at a dimer interface (52, 53), and the DcuS protein, which carries out a similar function in E. coli, has been proposed to act by a similar mechanism, in part via dimer interactions in the PAS domain (54).

Our proposal that EvgS may exist in three states (an inactive tight dimer, an active weak dimer, and an inactive weaker dimer or monomer, with the last being seen only in mutated forms of the protein) is supported by several lines of evidence. First, the large number of mutations found in the PAS domain that activate EvgS suggest that they are loss-of-function mutations at the structural level, and model building places many of them close to or at a putative dimerization interface in the PAS domain (27). In support of the latter point, the BvgS PAS domain has been shown to form a dimer when expressed as a soluble protein in E. coli (55). Second, we show here that releasing the cytoplasmic portion of the protein from its membrane anchor is sufficient to activate it. This suggests that some of the interactions forming the tight dimer are in the periplasmic domain (as shown by the structure) and the transmembrane domain and that without these, the cytoplasmic part of the protein forms mainly a weak, active dimer. However, expression of the cytoplasmic portion of the protein together with an S600I mutation in the PAS domain (which activates the full-length protein) leads to complete loss of activity even though the protein is still expressed, which is consistent with a further weakening of the weak dimer to an inactive state. AUC data on the cytoplasmic portion of the protein demonstrate the existence of a pH-regulated monomer-dimer equilibrium. As predicted by our model, there is a shift away from the dimer present at pH 7 when the protein is incubated pH 5.6, and this is also is seen if the protein contains the S600I mutation in the PAS domain.

We have not ruled out the possibility that a similar shift in a tight-weak dimer equilibrium in the periplasmic domain is also involved in enabling EvgS to respond to low pH in the periplasm, but two observations make this explanation less likely. First, no periplasmic mutations that give constitutive activation of EvgS have been found, despite nonbiased searches using three different selection methods (17, 27). Second, deletion of the periplasmic domain alone leads to an inactive protein (47) implying that the interactions responsible for the inactive state are predominantly in the transmembrane and cytoplasmic domains.

A recent detailed study on BvgS has generated data that support a model where a two-helix coiled-coil region immediately downstream of the BvgS PAS domain is crucial in mediating the activation of the kinase activity. According to this model, this well-conserved region may be affected by the state of the PAS domain itself, which could act as a “toggle switch,” changing the dynamics of the coiled coil from fairly rigid (in the phosphatase mode) to highly dynamic (in the kinase mode) (14). The authors of that study also mention the possibility that the PAS domain senses internal conditions in the cell, which hence may contribute to BvgS activity. The work we report here supports this view. The isolation of noninducible mutations in the periplasmic part of the protein has enabled us to clearly distinguish periplasmic and cytoplasmic detection of signal by EvgS, and by studying these mutations in the presence of the constitutive mutation S600I (which is necessary to give a high enough level of activity above background), together with the activity of the cytoplasmic part of EvgS alone, we have shown that induction of EvgS activity can be caused directly or indirectly by signals in the cytoplasm. This is an intriguing finding in the light of the recent demonstrations that Salmonella can detect a decreased cytoplasmic pH via the PhoP HKs, even when the external pH is neutral (56, 57). However, it poses a further functional puzzle, in that it has been shown that the decrease in the pH of the bacterial cytoplasm of E. coli when the external pH drops is only transitory, with a rapid restoration of neutrality (58, 59). Determination of how this transient drop might lead to sustained activation of EvgS-dependent gene expression will require detailed kinetic studies; the availability of the luciferase promoter probes which were used to obtain highly time-resolved data on induction of the members of the AR2 system should be useful here (26). These data also raise the intriguing possibility that EvgS has the ability to detect the pH gradient itself, which perhaps links back to the observations referred to above that it is affected strongly by oxidized ubiquinone.

In conclusion, our data are consistent with a model in which detection by EvgS of a ligand or ligands in the periplasm leads to structural rearrangements that, via H226 and P522, cause transduction of the inducing signal across the membrane. This signal, together with further information from the state of the cytoplasm, leads to a weakening of the dimer interface in the PAS domain, which in turn, probably through the mechanism described in reference 14, activates the autokinase activity of EvgS and strongly induces expression of the acid resistance AR2 genes together with several drug efflux pumps. It is not hard to see how such a mechanism may have a role in aiding the colonization of the gut by E. coli, but the nature of the ligand(s) remains unclear for the present.

## MATERIALS AND METHODS

### Bacterial strains and plasmids.

The bacterial strains and plasmids used in this study are listed in Table S1 at http://epapers.bham.ac.uk/3021/. HST08 supercompetent cells supplied with the In-Fusion hybrid cloning kit (TaKaRa-Bio) were used as the host for gene cloning. E. coli MG1655 Δ*evgS*::*cat ydeP-lacZ* Kan^r^ is the reporter strain for the acid induction assay (60). This strain was transformed either with the control plasmid pBAD24-his or with plasmids expressing wild-type or mutated versions of EvgS to determine the activity of the EvgS protein.

Bacterial cells were grown aerobically at 37°C in lysogeny broth (LB) (1% [wt/vol] NaCl, 1% [wt/vol] tryptone [BD Biosciences], and 0.5% [wt/vol] yeast extract) with added antibiotics (carbenicillin [100 μg/ml], kanamycin [100 μg/ml], or chloramphenicol [35 μg/ml]) as appropriate. For the acid induction assay, cells were grown in M9 medium (pH 5.6 or pH 7) supplemented with 100 mM KCl, 0.2% Casamino Acids (BD Biosciences), and 0.4% glucose. l-Arabinose (0.2%) was added to the culture at an optical density at 600 nm (OD_600_) of 0.1 to 0.2 to induce protein expression from the pBAD promoter.

### Plasmid construction.

All site-directed mutagenesis experiments were conducted using the QuikChange Lightning kit (Agilent) according to the manufacturer's instructions. To construct pBADEvgS-Cyt, codons for the cytoplasmic domains of EvgS (comprising amino acid residues 553 to 1193) plus a His_6_ C-terminal tag were PCR amplified from the plasmid pBADEvgS using primers 5′-GAGGAATTCACCATGGCGCTCAGTTCGTCGTCGT-3′ (forward) and 5′-CAAAACAGCCAAGCTTGTCATTTTTCTGACAGAAAAC-3′ (reverse). The amplified fragment was gel purified using the In-Fusion NucleoSpin (TaKaRa-Bio) kit and cloned into NcoI-HindIII-cut pBAD24 using the In-Fusion (TaKaRa-Bio) restriction-free cloning kit following the manufacturer's guidelines. The NcoI site was modified to ensure that the insert was in frame. To construct pET41c-EvgS-Cyt, the codons for the same fragment of EvgS were PCR amplified from the plasmid pBADEvgS using primers 5′-AAGGAGATATACATATGCGCTCAGTTCGTCGTCGT-3′ (forward) and 5′-CGCGTGGCACAAGCTTGTCATTTTTCTGACAGAAAAC-3′ (reverse). The amplified fragment was gel purified as described above and restriction cloned (NdeI-HindIII) into pET41c using the In-Fusion (TaKaRa-Bio) restriction-free cloning kit as described above. The plasmid pET41c used in our study is a modified version of its parent where the glutathione *S*-transferase (GST) tag has been replaced with C-terminal His_8_ tag. The resultant plasmid, pET41c-EvgS-Cyt, was transformed into BL21(DE3*) for overexpression and purification. The plasmid pET41c-EvgS-Cyt was also used as a template for mutagenesis to introduce the S600I mutation, using primers 5′-GTTCAAAAGCACTATTATGAATAATGACATTACCTTGCCAGTTTACAA-3′ and 5′-TTGTAAACTGGCAAGGTAATGTCATTATTCATAATAGTGCTTTTGAAC-3′, to enable purification of the cytoplasmic domain containing this activating mutation.

### Acid induction assay.

A single colony of each of the strains of interest was inoculated into 5 ml LB medium with the requisite antibiotics and grown overnight (10 to 12 h) aerobically at 37°C. The following day, cultures were diluted into supplemented M9 medium (pH 5.6 or pH 7) to an initial OD_600_ of 0.05, grown with shaking at 37°C to an OD_600_ of 0.1 to 0.2, and assayed for beta-galactosidase activity (61). All assays were done at least as biological triplicates.

### Overexpression and purification of the EvgS cytoplasmic domains.

BL21(DE3*)/pET41c-EvgS-Cyt was grown overnight aerobically in 50 ml of LB medium, pH 7. The following day, the culture was diluted into 800 ml of LB medium containing 100 μg/ml of kanamycin, to a starting OD_600_ of 0.05. The cultures were grown aerobically at 37°C to an OD_600_ of 0.3, and then the temperature was reduced to 24°C and the culture grown further to an OD_600_ of 0.4. Isopropyl-β-d-thiogalactopyranoside (IPTG) (0.1 mM) was then added to the cultures to induce expression of the protein, and the cultures were incubated overnight. They were then centrifuged at 6,000 rpm in JLA 8.1000 rotor (Beckman Coulter), and the harvested cells were resuspended in 100 ml of HEPES buffer (pH 7.4) (200 mM HEPES, 500 mM NaCl) containing a tablet of EDTA-free protease inhibitor (Sigma). The resuspended cells were lysed using an Emulsiflex-C3 homogenizer as per the manufacturer's guidelines. The lysate was centrifuged at 20,000 rpm using a JA 20 rotor (Beckman Coulter) and filtered through a 0.22-μm filter. The lysate was bound overnight to a 5-ml His-trap HP (GE Healthcare) column. The bound protein was eluted in HEPES buffer containing 500 mM imidazole (Sigma) and analyzed by SDS-PAGE. Fractions containing a band of the correct size were concentrated using a Vivaspin 20-ml concentrator (50,000 molecular weight cutoff [MWCO]) (GE Healthcare) and gel purified using an Akta Pure 25 (GE Healthcare LS) with a prepacked Hi-Load 16/600 Superdex 200 PG column. The cytoplasmic domain containing S600I was expressed and purified in the same way, except that cells were resuspended after harvest in 150 ml 150 mM HEPES–50 mM MOPS (morpholinepropanesulfonic acid)–200 mM NaCl (pH 7.4) and were eluted from the His-trap column in 150 mM MOPS–50 mM KOH plus 500 mM imidazole.

### AUC.

For analytical ultracentrifugation (AUC), sedimentation equilibrium runs were done using a Beckman XLA Optima after samples had been dialyzed extensively against 50 mM Tris-HCl buffer (pH 7 or pH 5.6). The dialysis buffer contained EDTA-free protease inhibitor and 2 mM phenylmethylsulfonyl fluoride (PMSF) (Sigma). After dialysis, protein solutions at an OD_280_ of 1 (corresponding to a protein concentration of 0.1 μM) were analyzed at 40,000 rpm and 20°C. A total of 200 scans were collected to capture the complete extent of the sedimentation of the protein samples at both pH values. The sedimentation distribution was analyzed using Sedfit (62, 63), and data were fitted using the following parameters: partial specific volume, 0.73000; buffer viscosity (poise), 0.01002. The sedimentation coefficient and mass distributions were extracted from the data to generate plots.

### Structural modeling.

To identify the best templates for the creation of the models, we used protein BLAST (BLASTp) against the PDB database. The dimeric structure of the periplasmic domain (PDB 4Q0C) of Bordetella pertussis BvgS (UniProtKB P16575) was identified as the top match to the EvgS query sequence. Structural alignments were performed using MAFFT (64) and visualized using ESPript 3 (65). EvgS homology models were built using I-TASSER (66). Output models had a C-score spread between 0.75 and 0.95. The C-score is a confidence score, the value of which varies from −5.0 to 2.0, with higher scores being indicative of high confidence in model building. Additional model optimization was performed manually using Coot as part of the CCP4 package (67). SSM superposition was used for structural alignment as implemented in CCP4. Similarity scores were calculated using SIM (http://web.expasy.org/sim/). Visualization of structural data was done with the PyMOL molecular graphics system, version 1.8 (Schrödinger, LLC.).
